# Guest Editorial: Climate Change: Healthy Solutions

**DOI:** 10.1289/ehp.115-a180

**Published:** 2007-04

**Authors:** Paul R. Epstein

**Affiliations:** Center for Health and the Global Environment, Harvard Medical School, Boston, Massachusetts, E-mail: paul_epstein@hms.harvard.edu

In 2001 the Intergovernmental Panel on Climate Change (IPCC; Houghton et al. 2001) concluded that climate is changing, humans are contributing, weather has become more extreme, and biological systems on all continents and in the oceans are responding to the warming. From the fourth IPCC assessment (Alley et al. 2007) and the Arctic Climate Impact Assessment (ACIA 2004), we now know that the deep oceans have accumulated 22 times more heat than has the atmosphere, ice melt is accelerating, wind patterns are shifting (that’s particularly ominous), and nonlinear surprises are very likely in store for the climate system and for the impacts on systems such as forests and coral reefs (Epstein and Mills 2005; Stern 2006). The implications for public health and well-being are daunting, as illustrated in articles throughout this month’s Environews section.

With weather turbulence turning heads on Wall Street, an emerging evangelical voice calling for “creation care,” the specter of “peak oil,” and a barrage of energy bills mounting Capitol Hill (the U.S. Congress), we appear to be on the verge of really taking the first steps toward confronting our energy budget. The goal of stabilizing atmospheric concentrations of greenhouse gases requires a 60–80% reduction of emissions over the coming few decades.

What follows are some considerations for crafting a comprehensive plan and some financial and policy instruments for implementation. Comparing life-cycle costs—the health, ecologic, and economic dimension—of proposed solutions can help differentiate safe solutions from those warranting further study, and from those with risks prohibiting wide-scale adoption. Solutions meeting multiple goals merit high ratings.

Energy conservation (demand side management) is clearly the first place to start. “Smart” urban growth; a smart grid (with optimizing meters and switches); hybrid vehicles; heat capture from utilities or “cogeneration” (two-thirds of produced energy is lost as heat); “green buildings”; and walking, biking, and improving public transport can get us halfway there—and save money.

Distributed generation (DG)—power produced near the point of use—with solar, wind, wave, geothermal heat pumps, and fuel cells can be fed into grids where they exist and, via “net metering” regulations, generate income for the individual producer. Where energy is scarce and grids are few, stand-alone systems—augmented with human power and stored in improved batteries—can pump water, irrigate fields, power clinics, light homes, cook food, and drive development. Clean DG also improves resilience in the face of more weather extremes (i.e., adaptation to climate change), reduces carbon emissions (i.e., prevention or mitigation of climate change), stimulates green industries, and creates jobs.

Biofuels hold a great deal of promise. However, converting corn to ethanol means less corn for animals and us, and may yield no net energy gain. Sugar ferments without adding energy (yeast suffices). But large plantations can deplete soils and groundwater and, in the Amazon, sugar for alcohol is pushing land clearing for soybean production deeper into the rainforest. In Indonesia, monoculture plantations of trees that produce palm oil are transforming and degrading vast swaths of prime forest, setting the stage for spreading fires and releasing biologically stored carbon from trees and peat. Cellulosic conversion of range grasses by microbe-generated enzymes may work, but land considerations still hold; recycling farm waste and garbage may yield the best results overall.

Building green buildings with healthy surroundings will create a critical syzygy, aligning clean energy production with sustainable forestry (e.g., bamboo) and green chemistry (which avoids petrolbased carcinogens in the manufacture of carpets, paints, furniture, fertilizers, and pesticides).

While it is unrealistic to think we can meet all of our energy needs without some fossil fuel use, natural gas is the cleanest burning and may be the best back-up source during the transition. Also, hydrogen gas (H_2_) can be separated from natural gas or methane (CH_4_) to use in fuel cells.

To implement such changes in our energy use, distribution, storage, and generation, corporations can change their products and practices (and many large ones, such as GE and Alcoa, have begun doing so). Financial institutions (e.g., bankers, insurers, managers of mutual and state pension funds) often have the longest time-line perspectives. Finance can be thought of as the central nervous system of the global economy: It is feeling the pain of huge losses from weather extremes, with insured losses rising from $400 million a year in the 1980s to $83 billion in 2005 (Epstein and Mills 2005), and they are cogitating on their response. Enlightened, self-interested actions of investors and insurers—through requirements for loans, influence on building codes, and reduced premiums for proactive directors and officers of firms, for example—could ripple through the entire global economy.

Governments must provide the incentives and create the infrastructure for the new economy. Credits for “clean tech” industries, progressive procurement practices (e.g., for hybrid fleets), and tax benefits for commercial models that defray upfront capital costs are among the incentives needed to launch infant industries and drive market shifts. Aligning rules, regulations, and rewards—and dismantling the enormous financial and bureaucratic disincentives—can help erect the necessary scaffolding for the low carbon economy.

Finally, the United States must sign the Kyoto Protocol (United Nations 1998). Under its umbrella, we can help create a substantive global fund for adaptation and mitigation that can make the clean energy transition a “win–win–win” for energy, the environment, and the global economy.

## Figures and Tables

**Figure:**
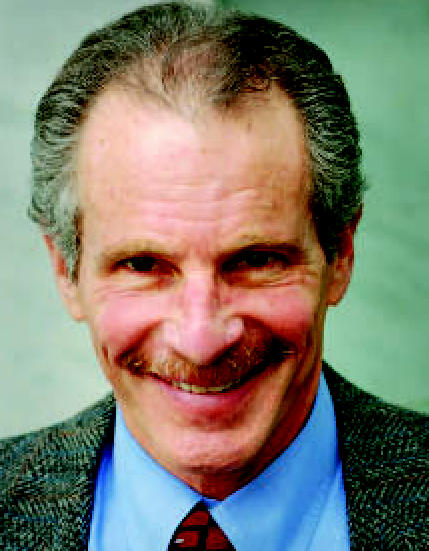
Paul R. Epstein

